# Strong Static Magnetic Fields Elicit Swimming Behaviors Consistent with Direct Vestibular Stimulation in Adult Zebrafish

**DOI:** 10.1371/journal.pone.0092109

**Published:** 2014-03-19

**Authors:** Bryan K. Ward, Grace X-J Tan, Dale C. Roberts, Charles C. Della Santina, David S. Zee, John P. Carey

**Affiliations:** 1 Department of Otolaryngology - Head & Neck Surgery, Johns Hopkins University School of Medicine, Baltimore, Maryland, United States of America; 2 Department of Neurology, Johns Hopkins University School of Medicine, Baltimore, Maryland, United States of America; 3 University of Pennsylvania School of Medicine, Philadelphia, Pennsylvania, United States of America; 4 Department of Biomedical Engineering, Johns Hopkins University School of Medicine, Baltimore, Maryland, United States of America; 5 Department of Neuroscience, Johns Hopkins University School of Medicine, Baltimore, Maryland, United States of America; 6 Department of Ophthalmology, Johns Hopkins University School of Medicine, Baltimore, Maryland, United States of America; University Zürich, Switzerland

## Abstract

Zebrafish (*Danio rerio*) offer advantages as model animals for studies of inner ear development, genetics and ototoxicity. However, traditional assessment of vestibular function in this species using the vestibulo-ocular reflex requires agar-immobilization of individual fish and specialized video, which are difficult and labor-intensive. We report that using a static magnetic field to directly stimulate the zebrafish labyrinth results in an efficient, quantitative behavioral assay in free-swimming fish. We recently observed that humans have sustained nystagmus in high strength magnetic fields, and we attributed this observation to magnetohydrodynamic forces acting on the labyrinths. Here, fish were individually introduced into the center of a vertical 11.7T magnetic field bore for 2-minute intervals, and their movements were tracked. To assess for heading preference relative to a magnetic field, fish were also placed in a horizontally oriented 4.7T magnet in infrared (IR) light. A sub-population was tested again in the magnet after gentamicin bath to ablate lateral line hair cell function. Free-swimming adult zebrafish exhibited markedly altered swimming behavior while in strong static magnetic fields, independent of vision or lateral line function. Two-thirds of fish showed increased swimming velocity or consistent looping/rolling behavior throughout exposure to a strong, vertically oriented magnetic field. Fish also demonstrated altered swimming behavior in a strong horizontally oriented field, demonstrating in most cases preferred swimming direction with respect to the field. These findings could be adapted for ‘high-throughput’ investigations of the effects of environmental manipulations as well as for changes that occur during development on vestibular function in zebrafish.

## Introduction

Several migrating vertebrate species use magnetic field cues for orientation; however, the sensory physiology and anatomic substrate of this sense remains incompletely understood [1]. Current theories of magnetosensation include use of ferromagnetic particles coupled with cell membranes, chemical reactions of radical ion pairs, and electromagnetic induction [1], [2]. Previously, only elasmobranchs (sharks, skates, and rays) were thought to detect magnetic fields via electromagnetic induction using a specialized organ called the ampulla of Lorenzini [1]. We have recently demonstrated in humans that a prominent and readily measurable neurobehavioral response (nystagmus, an involuntary rhythmic movement of the eyes) consistently occurs in a strong magnetic field. Multiple lines of evidence suggest that this effect is due to a hydrodynamic force (Lorentz force) generated by the interaction of the static magnetic field with ionic currents flowing through the endolymph and entering the stereocilia of sensory hair cells in the vestibular labyrinth [3]. Interestingly, Wu and Dickman identified central vestibular neurons responding to magnetic fields in pigeons, and suggest that the signal may originate in the inner ear lagena [4], [5].

Zebrafish (*Danio rerio*) are freshwater fish that have grown in popularity as a model laboratory species for genetic and neurobehavioral studies. Though non-migratory, zebrafish can be trained to recognize weak magnetic fields [6], and have recently been found to have bimodal directional swimming preference in a weak magnetic field [7]. In this study, we investigate adult zebrafish behavior in strong static magnetic fields, with the long-term goal of developing a rapid assessment of vestibular function in adult zebrafish.

Currently, vestibulo-ocular reflex testing is the accepted standard for assessing the vestibular system in zebrafish [8]–[10]; however, experiments can be time-consuming and technically difficult in adult fish. In this study we show that normal wild type adult zebrafish exhibit consistent looping and rolling behavior throughout exposure to a strong, static vertical magnetic field. We propose a potentially simple, clear assay of a behavioral response to a strong static magnetic field with a technique that does not require manipulation of individuals. This “no touch” technique could allow high-throughput investigations of the integrity of the vestibular apparatus in zebrafish suspected of having abnormal vestibular function because of mutations or chemical toxicity. Moreover, the technique could lend itself directly to investigations into the mechanisms by which vertebrates sense magnetic fields.

## Materials and Methods

### Ethics Statement

All procedures described in this research proposal were performed in accordance with a protocol reviewed and approved by the Animal Care and Use Committee at Johns Hopkins University (Protocol #FI12M324).

### Animals

*AB/AB* Zebrafish (15 adult pairs) were purchased from the Zebrafish International Resource Center (ZIRC, Portland, Oregon). The mean (±SD) standard length (i.e., distance from snout to base of caudal fin) was 2.5±0.2 cm, and fish were all 7.5 months old at the time of testing. Prior to magnetic field exposure, fish were maintained together in freshwater at 20–25°C on a 14/10 hour light/dark cycle. After magnetic field exposure, zebrafish were maintained in separate containers of fresh water for behavioral monitoring. No persistent post-exposure behavioral differences were seen in fish after brief exposures to the static magnetic fields. Fish were euthanized by ice water immersion at the conclusion of experiments [11].

### Behavioral Studies

We placed 30 *AB/AB* zebrafish (15 male/15 female) in 11.7 tesla (T) vertical and 4.7 T horizontal magnetic fields. Behavior was recorded inside and outside the magnet bore using an MRI-compatible video camera (Firefly MV, IEEE 1394a, Point Grey Research, Inc.) mounted above the container.

As vision can influence the motor response to any vestibular stimulation, we first sought to control for visual input. Zebrafish have photoreceptive visual pigments capable of sensing ultraviolet, blue, green, and red light, but they are not known to sense infrared (IR) light [12], [13]. Therefore, we used green illumination to test for vision-intact and IR illumination to test for vision-removed conditions. For all experiments, light was applied using a green or IR light-emitting diode (LED), adjacent to the camera. The camera was fixed approximately 6 cm above the fish, focused down at the fish from above.

To ensure lack of vision in our IR light conditions, 4 adult zebrafish were tested for optokinetic motor response behavior under the same IR illumination used inside the magnet. The fish were placed in a clear container of water surrounded by a drum with alternating black and white stripes (7.5°/stripe) on the inside of the drum. The drum was rotated about an Earth-vertical axis coaxial with the container. This experiment was done under green illumination and again under IR illumination. Video recordings made from above the container were assessed for rheotaxis (consistent swimming response against the direction of rotation).

For behavioral experiments in the vertical 11.7 T magnet, zebrafish were placed inside a non-reflective white, cylindrical plastic cup of 6.4-cm diameter. The cup was placed inside a lightproof box, and fish were observed by video for 1 minute in green light (peak wavelength 530 nm, spectral half-width 35 nm) and 1 minute in IR light (peak wavelength 950 nm, spectral half-width 42 nm). Each fish was then placed individually into the Earth-vertical 11.7 T magnetic field by lowering the cup containing that fish in water into the magnet bore from the top. Fish were briefly exposed to ambient fluorescent room light (<5 s) on transition into and out of the 11.7 T magnet. Once inside the magnet, ambient light was limited by the application of black felt to the bore entrance. The fish was observed for 1 minute in green light and 1 minute in IR light. Fish were then removed from the magnet, replaced inside the lightproof box, and observed again for 1 minute in both green and IR light.

To account for potential effects of sequence of light exposure on zebrafish behavior [12], [14], the protocol alternated green and IR light exposure for each fish, such that half the fish were exposed to visible green light upon initial entry into the magnet and half to darkness with IR light. In the cylindrical container used within the vertical magnet, a depth of 1.5 cm of water was used such that the water surface extended just above the fish's dorsal fin. The latter half of trials (n = 15) was performed using 3.0 cm of water to assess for differences in water column level on the response. To determine if the 11.7 T magnet's fringe field (≤0.004 T at ≥2 meters from the magnet bore) influenced swimming behavior, 10 fish were also observed in the fringe field and then later observed in Earth magnetic field (5.8×10^−5^ T).

Within 48 hours after exposure to the 11.7 T magnetic field, all 30 fish were then placed in a horizontal 4.7 T magnetic field to observe if directional swimming preferences could be elicited. The horizontal magnet's bore was larger (22 cm diameter), permitting a wider container for improved swimming behavior observation. Fish were therefore placed in a rectangular plastic container (10.2 by 10.5 cm dimensions), enclosed within a lightproof box and covered with a double layer of black felt to minimize ambient light. A 1.5 cm water column was used. Fish were observed for 30 s in IR light before entry, for 60 s within the center of the 4.7 T magnet, and for 30 s immediately after exposure to the magnet. The fringe magnetic field in which the fish remained for the 30 s pre- and post-exposure conditions was approximately 0.008 T. The middle 30 s time interval of video from inside the magnet was analyzed, excluding video during container translation into and out of the bore so as to limit motion artifacts induced by container movement. IR light conditions were used throughout this paradigm to avoid confounding effects of vision on heading preference. The larger 4.7 T magnet bore accommodated the lightproof box, therefore excluding any ambient light exposure during the transition into or out of the magnet. Fish (n = 10) were also observed in Earth ambient magnetic field away from the magnet fringe field. Water temperature for all experiments was between 21–23°C.

All video data collection was performed at a rate of 30 frames per second using Point Grey software and stored on a laptop computer. Fish location and heading direction were extracted semi-automatically using custom-developed Matlab object tracking software (Mathworks, Natick, MA). For IR conditions in the vertical magnet, poor contrast frequently required manual marking of fish location. The fish's center of mass was marked every 5 frames for each video. Average fish swimming velocity for each trial was calculated by dividing total path length by video duration. Counts of swimming paths that transected the central 40% of the horizontal area of the container were assessed for each trial in the horizontal magnet by demarcating a rectangular region of interest set 100 pixels from edges of frame limit, corresponding to approximately 1.67 cm from the tank edge.

### Lateral line ablation

Because prior studies have suggested a role for the lateral line in magnetosensation, [15], [16] a subpopulation of fish was treated with 0.002% gentamicin sulfate immersion and then replaced into the vertical magnetic field to assess for the influence of the lateral line on the observed behavior. After all 30 fish were exposed to both vertical and horizontal static magnetic fields and observed for one week, a cohort of 10 fish was placed in a well-aerated bath of 0.002% gentamicin sulfate (100 mg/mL, Butler Schein, Dublin, OH) for 8 hours to ablate both lateral line canal and superficial neuromast function [17] before re-exposure to the magnetic field. A second cohort of 10 control fish simply underwent repeat exposure to the magnetic field. Within 6 hours of gentamicin exposure, the treated fish were replaced in the 11.7 T magnet to assess for changes in magnet-induced behavioral responses that could be attributed to injury to lateral line hair cells. Short-term immersion in gentamicin is not believed to damage inner ear hair cells in cichlid fish [18], and while the short-term effects of immersion on inner ear hair cells of the zebrafish in particular are unknown, inner ear damage from gentamicin typically occurs over several days after administration [19], [20], including in adult teleost fish [21]. Any inner ear vestibular lesion, if present, would therefore be expected to be incomplete during the short time course between administration and testing. Following behavioral assessment (and thus approximately 12 hours after gentamicin exposure) DAESPI (2-(4-(dimethylamino)styryl)-Nethylpyridinium Iodide, Invitrogen Molecular Probes, Eugene, OR) was applied to enable visualization of lateral line hair cells by immersing gentamicin-exposed and control fish in a 0.08% solution for one hour. Fish were then euthanized and examined under fluorescent microscopy (Nikon SMZ 1500 stereomicroscope). Photographs were captured using a digital microscope camera (ProgRes MFcool) with a GFP filter set.

### Statistical analysis

Data were analyzed using Stata 12 (Stata Corp, College Station, TX). After confirmation that the distributions of swim velocities were normal, analysis of variance was performed for assessing group differences before, during and immediately after magnetic field exposure. Pre-planned comparisons were intended between fish swim velocities inside and outside of the magnetic field. Between-group comparisons were performed if significant using paired *t*-tests. Paired t-tests were also used to compare swimming velocity inside the magnet for green and IR light. Univariate analysis was used to assess relationships between body mass, length, and swimming velocity inside the magnetic field. Values were considered statistically significant for a two-sided test with p-value <0.05.

## Results

### IR illumination eliminates optokinetic response

Zebrafish (N = 4) in a clear plastic container placed inside a rotating drum with alternating black and white stripes all exhibited rheotaxis, a consistent swimming response against the direction of drum rotation, under green-light illumination. In contrast, no vision-dependent response was elicited in 950-nm peak wavelength IR light **(See Video S1 online**). These results confirmed that IR illumination effectively removed vision for those parts of the magnetic experiments in which it was the only illumination used.

### Strong vertical magnetic field causes rapid circling, rolling and diving

**Figure 1:**
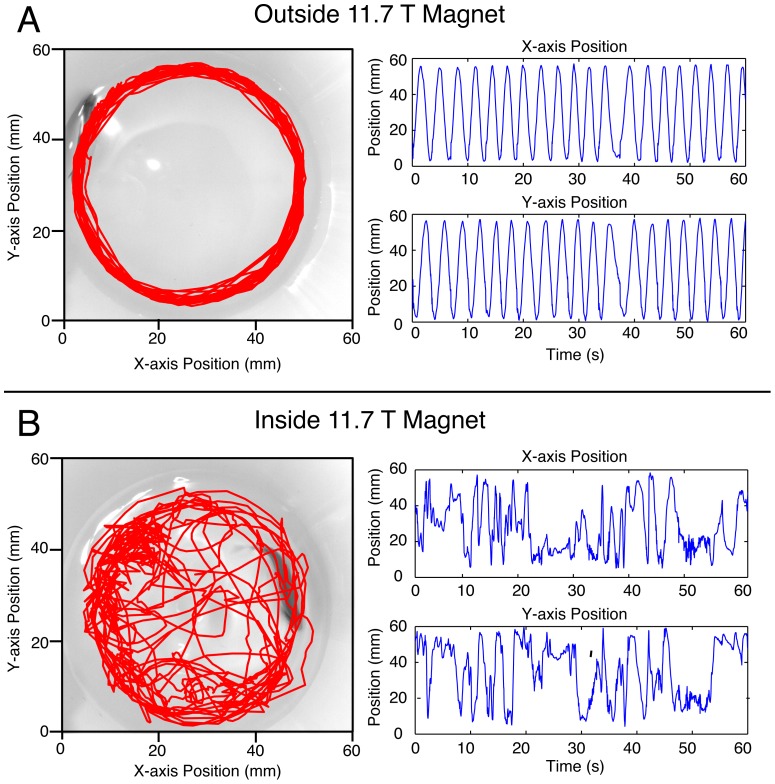
Adult zebrafish behavior outside and inside of an 11.7 T vertical magnetic field. Tracing of adult zebrafish path in visible green light during 1 minute prior to magnetic field entry (a) and during 1 minute inside the magnet (b). X- and y-position coordinates are displayed as a function of time. Upon entry into the magnet, fish swimming becomes erratic, with frequent rolling, tight circling and increased swimming velocity.

Upon entering the 11.7 T static vertical magnetic field, average zebrafish swimming velocity significantly increased (*F_2, 89_*, = 15.7, p<0.001). Twenty of 30 (66%) adult zebrafish demonstrated either increased swimming velocity and/or erratic rolling behavior. The direction of swimming movements was not consistently clockwise or counterclockwise; rather, affected fish frequently changed direction inside the magnetic field. **Figure 1** and **video S2** demonstrate a typical example of normal swimming behaviors before entering the magnet and altered swimming behavior inside the strong static magnetic field.

Half of the fish developed postural instability inside the vertical magnet, remaining predominantly on their side or rolling continuously in the magnet (15/30, 50%). Fish that rolled commonly exhibited a preferred roll direction, i.e., some rolled with the dorsal fin toward the right >90% of the time while in the magnet, and others rolled toward the left >90% of the time while in the magnet. Only two fish that rolled did not display a clear directional preference for rolling. No associations were identified between either fish length or body mass and swimming velocity in the magnet (p = 0.48 and p = 0.77, respectively).

Water column height did not influence the presence of postural disturbance or of increased swimming velocity inside the magnet (p = 0.59). When comparing zebrafish swimming behaviors in the fringe field of the vertical magnet (0.008 T) to those in Earth magnetic field (5.8×10−5 T), no significant differences in behavior were observed; however, there was a trend toward increased swimming velocity in the fringe field (8.1 mm/s ± 4.1 vs. 5.2 ± 3.4 mm/s, p = 0.06).

### Magnetic effects on orientation and locomotion are not suppressed by a static visual surround

**Figure 2:**
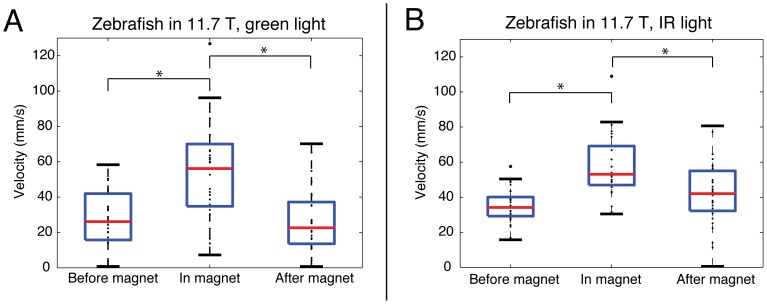
Box plots of average swimming velocity for all 30 fish before, during, and after entry into the magnetic field. *, Significant increases in swimming velocity are seen inside the magnet in both green visible light (a) and in darkness with invisible infrared illumination (b), p<0.001.

**Figure 3:**
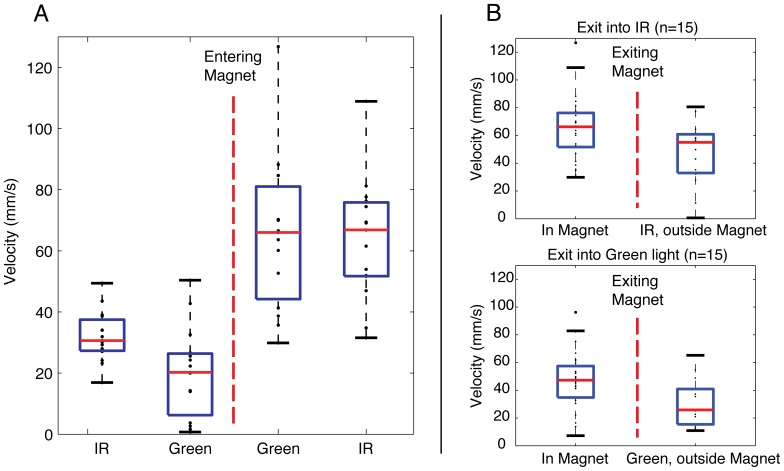
Box plots of adult zebrafish average swimming velocity demonstrating effect of vision on behavior outside and inside of an 11.7 T vertical magnetic field. X-axis demonstrates the order of light exposure from left to right. Zebrafish were observed for one minute in each lighting condition. The red, dashed vertical lines represent a transition into or out of the 11.7 T vertical magnet. a) Upon entering the magnetic field, swimming velocity increases. There was, however, no change in swimming velocity when lighting in the magnetic field changed from green (visible) to IR (invisible). b) Box plots are shown comparing mean swimming velocities during two minutes inside the magnet to the first minute after exiting the magnet. For those fish that transitioned out of the magnet in IR light (n = 15, top panel), there was less of a decrease in swimming velocity compared to those fish that transitioned out of the magnet in green light (n = 15, bottom panel). IR, infrared.

To determine whether vision affected the behavioral response, fish were observed in both green (visible) and IR (invisible) light before, during, and immediately after exposure in the magnet. In IR light outside the vertical magnet bore, each fish slowly swam in a consistent direction around the perimeter of its circular container. In green light outside the magnet, fish changed directions more frequently and swam slower than in IR light. Despite behavioral differences outside the magnet, however, fish demonstrated similar rolling behavior inside the magnetic field regardless of lighting conditions, including increased swimming velocity (*F_2, 89_*, = 11.7, p<0.001, **Fig. 2**). The effects of vision on behavior inside the magnet were minimal, with no significant differences in observable behavior or in swimming velocity between green and IR light conditions (p = 0.58, Fig. 3a). Immediately after removal from the magnetic field, all fish maintained upright posture, regardless of light conditions.

Since the order in which fish were exposed to light may influence behavior, the pattern of light exposure was varied such that half the fish were transitioned out of the magnet in green light and half were transitioned out of the magnet in IR light. When separating out the order of light exposure, fish that were moved out of the magnet in green light had swimming velocity immediately after exiting the magnet that was similar to that seen before entering (p = 0.54). Those fish that remained in IR light upon exit from the magnet, however, maintained increased average swimming velocity over the one minute after exiting compared to before entry (p = 0.012). **Figure 3b** shows a smaller decrease in swimming velocity for those fish transitioned out of the magnet in IR light compared to those transitioned out of the magnet in green light.

### Strong horizontal magnetic field influences heading direction preference

**Figure 4:**
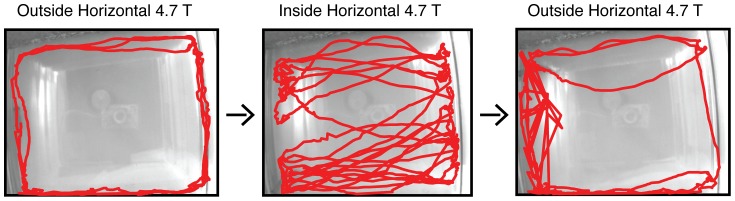
Example position versus time trace of fish in a horizontal 4.7 T magnet. The red line identifies fish position during 30's perimeter; whereas inside the magnet, fish cross the center more frequently, increasing speed along a preferred bidirectional heading direction.

Several fish that consistently rolled in the vertical magnetic field also dove in the direction of the magnetic field vector (**see Video S2 online**). Given limitations of magnetic field orientation and container dimensions for determining heading preference in the vertical magnet, a horizontal 4.7 T magnet was used in the second experimental paradigm to determine if there was a change in heading preference caused by the magnetic field vector. Each fish was replaced in a horizontally oriented 4.7 T magnet in darkness with IR light. A typical example of altered swimming behavior in the horizontal magnetic field is shown in **figure 4** by the position trace of the fish over time before, during and after exposure in the 4.7 T magnet (**see Video S3 online**). Similar to the behaviors observed in the vertical magnet, we saw postural disturbances of some fish in the horizontal magnet, with 10 fish (33%) rolling ∼90° about the long axis of the fish's body, a behavior that was never observed outside of the magnet bore. In the horizontal magnet, this behavior occurred intermittently and primarily when the fish aligned its long axis with the magnetic field vector. Only two of 10 fish that rolled in the horizontal magnet also rolled in the vertical magnet, and 4/30 fish (13%) did not roll in either magnet.

**Figure 5:**
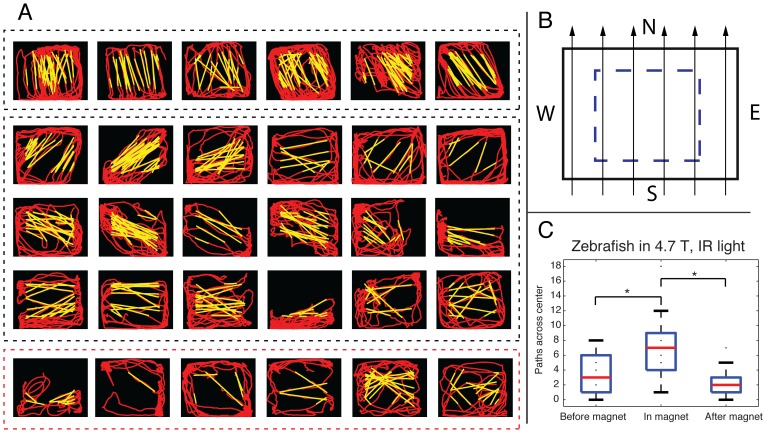
Adult zebrafish swimming behavior in a horizontal 4.7 T magnet in darkness with infrared illumination. a) Traces of zebrafish movement during a 1-minute time interval inside the magnetic field are shown in red and yellow. b) A region of interest (ROI, dotted blue line) in the center of the container was defined, and lines were fit to zebrafish swimming paths crossing the ROI (yellow lines in (a)). Red lines in (a) represent swimming paths during the 1-minute time interval inside the magnetic field that occurred outside the ROI or where no line could be fit across the ROI. The magnet north (N) and south (S) poles and magnetic field vectors are demonstrated in b. Most fish developed a heading preference inside the magnet, increasing velocity along its direction of preference. Preference varied by fish; however, the majority avoided the N/S magnetic field vectors. Some fish in the 11.7 T vertical magnet frequently dove in the direction of the magnetic field vector (a, *top row*); these fish preferred the N/S magnetic field vectors in the 4.7 T horizontal magnet. Six fish (*bottom* row) showed no consistent heading preference. c) Box plots showing the number of times a fish crossed the container's central zone over a 30-s interval are shown. Even while kept in darkness (with IR) throughout transit into and out of the horizontal magnet, fish more frequently crossed the central zone while in the magnet than while outside the magnet, suggesting that they normally can detect directional cues in a strong magnetic field without visual input. * indicates statistical significance at p<0.01

The majority of fish (n = 24, 80%), however, demonstrated heading preference in the horizontal magnet, consistently increasing swimming velocity along a particular axis relative to the magnetic field axis. Position traces for each fish inside the horizontal magnet are shown in separate panels in **Figure 5**. Zebrafish that consistently attempted to dive inside the vertical 11.7 T magnetic field more commonly exhibited preference for the N/S heading direction while in the horizontal magnet (highlighted in **Fig. 5a** **,** ***top row***). The majority of fish (25/30, 83%) also crossed the container's center (**Fig. 5b** **,** ***dashed rectangle***) more frequently than when outside the magnet (*F_2, 87_*, = 8.6, p<0.001, **Fig. 5c**).

After initial assessment of all fish in both magnetic fields, fish were maintained separately for identification and monitoring, and cohorts were replaced in the magnetic fields one week later. Rolling behavioral responses for control fish were repeatable on the same testing day (n = 4) and one week later (n = 10).

### Absence of lateral line hair cells does not ablate altered behavior in a strong vertical magnetic field

**Figure 6:**
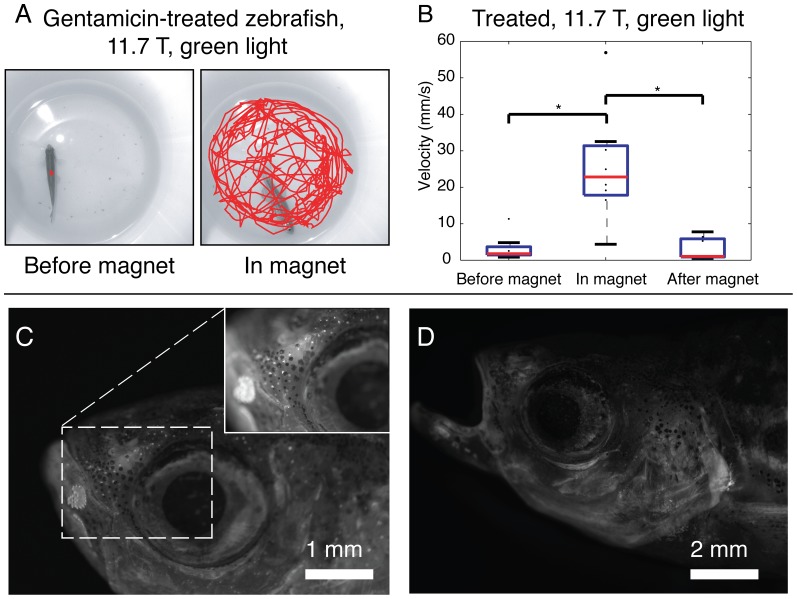
Effects of 0.002% gentamicin sulfate immersion. a.) 1-minute tracing of swimming path of adult zebrafish treated with gentamicin immersion before and during exposure in 11.7 T vertical magnet. Despite remaining stationary outside the magnet (left panel), erratic rolling is observed in the magnet (right panel). b.) Box plots of average swimming velocity by test condition for 10 treated fish. Treated fish demonstrate greatly increased swimming velocity inside the 11.7 T magnet. Examples of DAESPI lateral line staining of the head from control (c) and treatment (d) groups. Images were captured one hour after DAESPI staining and immediately after euthanasia. Intact lateral line neuromasts were seen in control fish and in none of the treated fish.

Gentamicin immersion treated fish demonstrated altered swimming behavior outside the magnet compared to pre-treatment, preferring to remain stationary, and showing decreased rheotaxis. An example of this decreased movement over a 1-minute interval is shown in **figure 6a** ‘Before Magnet’ (see comparison of **figure 6b** to **figure 2a** for group swimming velocity before and after gentamicin immersion). Inside the vertical 11.7 T magnet, however, a striking disturbance of behavior was again noted (**Fig. 6a**). Swimming velocity significantly increased inside the magnet for both green light (*F_2, 23_*, = 15.9, p = <0.001, **Fig. 6b**) and IR light conditions (*F_2, 23_* = 6.5, p = 0.0063). The same fish (n = 6) that rolled continuously in the magnet prior to treatment also rolled post-treatment.

To confirm that the neuromasts were ablated, fish were immersed in DAESPI for one hour, euthanized, and observed under fluorescence microscopy. Seven treated fish underwent this vital dye staining. Superficial and canal neuromasts were readily identified in control fish (n = 3). No viable neuromasts were identified in fish after gentamicin immersion (**see example in** **Fig. 6c**).

## Discussion

We have shown that strong static magnetic fields (4.7–11.7 T) profoundly disturb the orientation and locomotion behaviors of adult zebrafish, and the independence of these effects from other sensory modalities suggests that they are mediated by the vestibular system. Fish use visual cues, proprioceptive signals, including from their lateral line, and labyrinthine sensation to maintain posture. The striking effects that strong static magnetic fields had on swimming and posture did not depend on vision, as we saw no significant behavioral differences inside the magnet among the responding fish between light and dark conditions. While adult fish with chemically ablated lateral line function appeared to exhibit diminished swimming velocity outside of a magnetic field, they are capable of otherwise swimming normally [22], and still demonstrate the same postural disturbances in the magnetic field. The persistence of the same behavioral alterations after removing visual and neuromast inputs suggests that this unusual behavior is mediated by another sensory system.

Similar rolling and looping behaviors have been described in fish upon entering microgravity environments [23], after surgical removal of the otoliths [24], and in adult fish with hair cell defects [25], supporting altered labyrinthine sensation as a potential source for the similar behaviors induced by strong magnetic fields in this study. We found further support for a vestibular basis for the altered swimming behaviors induced by strong magnetic fields by observing higher swimming velocities after exposure in the magnet for fish transitioned out of the magnet in darkness, than for those fish transitioned out of the magnet in visible green light (**figure 2b**). This finding is consistent with characteristics of the velocity storage mechanism known to occur in the vestibular reflexes. Velocity storage is a well-recognized phenomenon in mammalian vestibular physiology that has also been identified in teleost fish [8], [26], [27]. Although its function is thought to enhance responses to low-frequency stimuli from the semicircular canals [28], [29], it is usually manifest after rotating in darkness, as both a persistent perception of rotation and nystagmus (rhythmic movement of the eyes) that persists beyond what is predicted by movements of inner ear endolymph alone. Robinson proposed that velocity storage could be accomplished by a feedback loop operating in a circuit including the vestibular nuclei [28]. Lesion studies in monkeys suggest that velocity storage arises from neurons in medial vestibular nucleus (MVN) and descending vestibular nucleus (DVN) whose axons cross the midline [30]. This perseveration of vestibular nystagmus due to velocity storage is also suppressed by visual fixation in light. The finding of a persistent behavioral effect in darkness following removal from the magnetic field may represent another form of velocity storage in response to a labyrinthine stimulus that occurred while within a strong magnetic field.

While ferromagnetic or paramagnetic particles have not been discovered in the inner ears of *Danio rerio*, diamagnetic forces acting on the irregularly-shaped otolith organs could induce altered graviception and postural instability [31]. This hypothesized effect on the otolith organs is consistent with current theories of altered postural stability in fish within microgravity environments, in which the gravity-dependent otolith organs become unreliable environmental sensors [32], [33]. Diamagnetic forces are not sensitive to magnetic field polarity. In a magnetic field gradient, a diamagnetic particle such as an otolith surrounded by endolymph will be equally repelled from either magnetic pole, however, in a homogeneous magnetic field like the ones used in this study, translation forces are absent, and torque on an irregularly shaped otolith would predominate. Torque applied to an otolith in the magnetic field could induce altered posture, and may also contribute to the observed consistent rolling behavior of each fish to a preferred side, dependent on subtle otolith asymmetry [32], [34].

Static magnetohydrodynamics (MHD) is a newly proposed labyrinthine mechanism of magnetic field-induced magnetosensation that requires only a static magnetic field and an ionic current to produce a Lorentz force within a conductive fluid. The interaction of normal ionic endolymph currents summed up over many hair cells arrayed in a sheet in the utricular macula with the very strong static magnetic field produces sufficient hydrodynamic force to move the overlying endolymph (see Figure 4 in Roberts et al. 2011). This endolymph motion is in the direction needed to stimulate primarily the horizontal semicircular canals and may account for an observed nystagmus in humans [3]. This mechanism may also induce circling behavior in mice after magnetic field exposure [35]. A Lorentz force mechanism for magnetosensation is polarity-sensitive, providing additional heading information over transduction using ferromagnetic, paramagnetic, or diamagnetic properties of tissues. Polarity-dependent postural disturbances have additionally been identified in rats introduced to strong static magnetic fields [36].

Unexpectedly, we identified differences in behaviors of individual fish dependent upon the magnetic field vector orientation with respect to the fish's body. When exposed to a strong static vertical magnetic field (i.e. while in the vertical magnet bore, in which the magnetic field vector is directed along the fish's dorsoventral axis when the fish is upright), half the population of tested fish failed to maintain upright posture, whereas the others either increased swimming speed or swam normally. Fish that seemed unperturbed by the vertical magnet showed strong behavioral changes in the *horizontally* oriented magnet, either by frequently rolling when their body's long axis aligned with the magnetic field vector or by exhibiting a strong directional heading preference. While differences in magnet field strengths between the horizontally and vertically oriented magnets limits behavioral comparisons, all the adult fish studied experienced dramatically altered behavior within a strong magnetic field. The observed behavioral variations could represent small anatomic differences in labyrinthine orientation or differences in magnetic field preferences determined by genetic group [7].

While labyrinthine forces may contribute to this perceptual disturbance, we cannot exclude other non-labyrinthine sensorimotor contributions. Magnetophosphenes, for instance, are well-known visual phenomena that can occur inside magnetic fields, independent of vision [37], and could induce altered behavior in zebrafish. Furthermore, magnetite crystals have been described in the olfactory lamellae of trout [2], [38]. If present in zebrafish, these crystals would be strongly affected by the magnetic fields used in this study; nevertheless, magnetite crystals have not yet been reported in zebrafish.

As an increasingly important model species in genetic and behavioral analysis, zebrafish are an excellent organism for better understanding mechanism of magnetosensation. Though the magnetic fields used in these experiments exceed ambient Earth magnetic field by about 5 orders of magnitude, the finding of clear behavioral responses in adult zebrafish to a static magnetic field should contribute to future studies investigating mechanism of transduction of a recently discovered magnetic sense in humans [3]. The identification of a vestibular mechanism for this behavior would also provide a novel, ‘no-touch’ neurobehavioral assay for vestibular function in zebrafish and could lend itself to high-throughput screening as it is relatively fast, easily observed, and readily quantified, features that are favorable for automation.

The strengths of the magnetic fields used in this study were of similar magnitude to those used when obtaining magnetic resonance imaging (MRI) studies in humans. The finding that all humans with intact labyrinthine function have a measurable neurobehavioral response (nystagmus) while in an MRI magnet with sufficient field strength [3] suggests that the results of these studies in zebrafish may have broad implications for understanding not only magnetosensation but also effects on humans exposed to strong static magnetic fields during magnetic resonance imaging.

## Supporting Information

Video S1 **Example of adult zebrafish optokinetic response behavior to a rotating drum of black and white stripes in the two lighting conditions used in these experiments.** In visible green light, the fish demonstrates robust optokinetic motor response to an opkinetic drum stimulus, aligning so as to swim in opposition to the perceived motion of the visual surround. In darkness with infrared illumination, the fish appears to be unaware of the rotating vertical stripes, thus confirming poor visual acuity under infrared-only lighting.
(MPG)

Video S2 **Example of adult zebrafish behavior in visible green light before, during, and immediately after exposure to the center of an 11.7 T vertical magnet.** The fish circles the container outside the magnet. Inside the magnet, the fish frequently rolls and attempts to dive. No behavioral differences are observed after removing the fish from the magnet compared to before magnet entry.
(MPG)

Video S3 **Behavior of the same adult fish in Video S2 (** **Figure 5** **, top row, 4^th^ panel), in infrared light before and during exposure to 4.7 T horizontal magnetic field.** Prior to magnet entry, the fish slowly swims around the container's perimeter in darkness. Inside the magnet, the fish develops a heading preference for approximate N/S magnetic field vectors, increasing swimming velocity along this path and avoiding long swim paths in the E/W directions.
(MPG)
